# Spinal and bulbar muscular atrophy in a patient with Parkinson's disease – Case report

**DOI:** 10.1016/j.prdoa.2026.100484

**Published:** 2026-07-21

**Authors:** Alexander Grotemeyer, Julie Rösch, Juergen Winkler, Martin Regensburger

**Affiliations:** a Department of Molecular Neurology, University Hospital Erlangen, Friedrich-Alexander-Universität Erlangen-Nürnberg, Erlangen, Germany; b Department of Neurology, University Hospital Erlangen, Friedrich-Alexander-Universität Erlangen-Nürnberg, Erlangen, Germany; c BDT Institut für Bildgebende Diagnostik und Therapie, Erlangen, Germany

**Keywords:** Spinal and bulbar muscular atrophy, Parkinson's disease, Dropped head syndrome

## Abstract

Spinal and bulbar muscular atrophy and Parkinson's disease rarely coexist. We describe a male patient presenting with parkinsonism, hyposmia, with DAT-SPECT deficits, responsive to dopaminergic therapy, preceding recognition of SBMA confirmed genetically. Dopamine agonist–associated dropped head syndrome unmasked underlying lower motor neuronopathy, highlighting diagnostic pitfalls and overlap between synucleinopathy and polyglutamine disease.

Spinal and bulbar muscular atrophy (SBMA, Kennedy's disease) is an X-linked adult onset, lower motor neuronopathy caused by a CAG expansion in the androgen receptor (AR) gene [1]. There is a single report of concurrent SBMA and neuropathologically confirmed multiple system atrophy of the cerebellar subtype (MSA-C) [2]. Postural tremor may occur in SBMA, but presynaptic dopaminergic degeneration with motor symptoms related to concurrent Parkinson's disease (PD) has not been described.

We here report a man who in his late 50s presented with new-onset asymmetric parkinsonian symptoms, which initially dominated the clinical picture and prompted PD evaluation.

Neurological examination showed decreased right arm swing, right-dominant bradykinesia with impaired fine motor movements, right-dominant postural tremor of the upper limbs, fine motor slowing, and mild gait unsteadiness. Olfactory testing revealed hyposmia (6/12) and a subsequent DAT-SPECT demonstrated an asymmetric presynaptic dopaminergic deficit, with reduced specific binding ratios in the striatum (right = −0.79, left = −1.92) and putamen (right = −1.28, left = −2.53), most pronounced in the left putamen (Fig. 1A). Baseline UPDRS III was 14 and improved to 5 points upon initiation of dopaminergic medication. Treatment included rasagiline (1 mg per day) and piribedil (150 mg per day). The clinical response mainly supported a dopaminergic component of bradykinesia and parkinsonism. Hence, a diagnosis of PD was made.

**Fig. 1:**
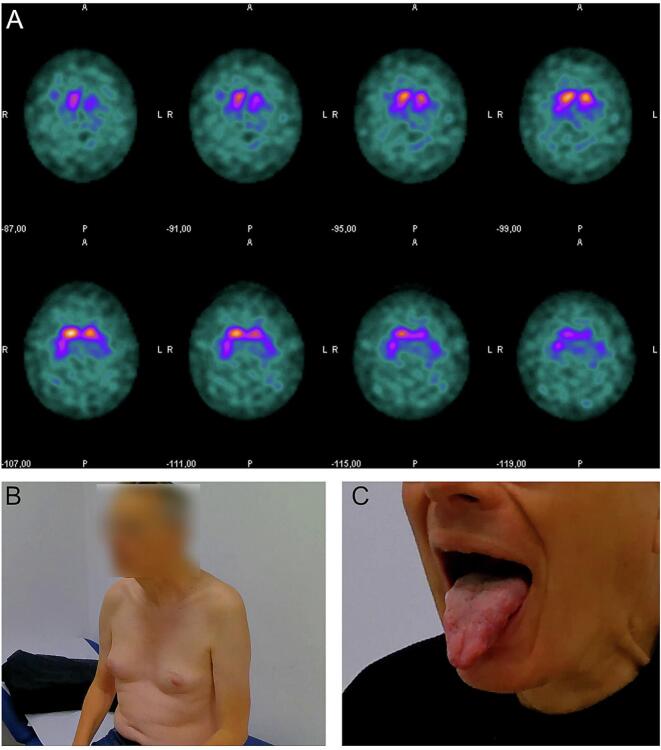
(A) Dopamine transporter imaging revealed reduced signal binding ratio of the left striatum and putamen. (B) Aspect of antecollis and gynecomastia. (C) Tongue atrophy.

At the time of PD diagnosis, the patient did not report longstanding postural tremor or muscle cramps. However, retrospectively, the patient reported slowly progressive dysarthria and dysphagia with daily choking for 1–2 years *before* the onset of PD motor symptoms. These bulbar symptoms were initially mild and clinically overshadowed and misinterpreted as potentially PD-related. These symptoms did not show clinically relevant improvement under dopaminergic therapy. Three years after the onset of PD symptoms onset and 8 months after initiation of piribedil, he developed a rapidly progressive dropped-head syndrome. The dropped head was passively correctable, associated with neck extensor weakness MRC 3/5 (Fig. 1B–C), and without clear signs of fixed cervical dystonia. EMG revealed fasciculations in multiple regions on 3 of 4 levels, chronic neurogenic changes in the left semispinalis capitis muscle and the right biceps brachii muscle, along with widespread lower motor neuron signs. There was no clinical evidence of ocular fatigability or fluctuating ptosis/diplopia. The electrophysiological myasthenia and myositis work-up did not reveal evidence for these aetiologies. Levels of CSF neurofilament light chain (NfL; 1476 pg/mL; reference <829) and serum creatine kinase (CK; 937 U/L; reference <190) were elevated. CK elevation supported skeletal muscle/lower motor neuron involvement whereas NfL was interpreted as a non-specific marker of neuroaxonal injury in this setting. It is important to note that neither of the two parameters is disease specific. Tapering of the dopamine agonist led to progressive improvement of dropped head syndrome over five months, consistent with prior reports [3]. CK levels showed only a partial decrease after tapering of piribedil, as they remained elevated at 724 U/L.

Further neurological examination at this time point revealed asymmetric proximal weakness (left predominant), proximal muscle atrophy of both arms, areflexia, pectus excavatum, gynecomastia, facial weakness, and fasciculations of face and tongue muscles. Thus, weakness was not restricted to the neck but also involved proximal limb muscles. Also, in this context, family history was reported by the patient as negative for similar symptoms or known neuromuscular disease. However, no relatives were personally examined, and subtle manifestations in family members cannot be excluded, retrospectively. Importantly, the patient had no children. The suspected diagnosis of SBMA was confirmed by genetic testing which showed a hemizygous CAG repeat expansion (42 ± 1 repeats) within the *AR* gene. Cardiological examination was unremarkable, and there were no autonomic symptoms.

To the best of our knowledge, this is the first description of genetically confirmed SBMA in a patient with PD. PD symptoms were the dominant presentation at onset, whereas SBMA manifestations became more clinically evident during dopaminergic therapy. This observation expands the spectrum of SBMA co-pathologies. It shows that α-synucleinopathy and polyglutamine-mediated lower motor neuronopathy may manifest in close temporal association. The attribution of individual symptoms was challenging. Asymmetric parkinsonism, hyposmia, DAT-SPECT abnormality, and dopaminergic responsiveness supported PD, whereas bulbar symptoms, tongue fasciculations, proximal atrophy, elevated CK, neurogenic EMG changes, and *AR* CAG-expansion supported SBMA. The postural tremor may have reflected PD, SBMA [4], [5], or both, although the absence of a clear history of longstanding postural tremor or muscle cramps before PD onset did not support a definite prodromal SBMA tremor syndrome. The rapid onset and improvement of dropped-head syndrome after piribedil tapering argue against SBMA progression alone. We therefore consider dropped head syndrome a likely multifactorial manifestation, possibly facilitated by underlying SBMA-related neuropathic axial weakness and aggravated by dopaminergic therapy. While the reported combination appears exceptionally rare, awareness of such presentations may help to avoid misinterpretation of symptoms and guide consideration of rare comorbidities. This may be particularly relevant when parkinsonian symptoms coexist with bulbar symptoms resistant to dopamine therapy, fasciculations, unexplained CK elevation, or disproportionate axial weakness.

## Relevant financial disclosures

The authors declare no financial disclosures relevant to the manuscript.

## Ethical compliance statement

Ethical approval was not required for this work, since it retrospectively describes a single patient case. Written informed consent was obtained from the patient for case presentation in written and video format. All authors confirm that they have read the Journal's position on issues involved in ethical publication and affirm that this work is consistent with those guidelines.

## CRediT authorship contribution statement

**Alexander Grotemeyer:** Writing – original draft, Investigation. **Julie Rösch:** Resources, Writing – review & editing. **Juergen Winkler:** Writing – review & editing, Investigation. **Martin Regensburger:** Writing – review & editing, Investigation.

## Declaration of competing interest

The authors declare that they have no known competing financial interests or personal relationships that could have appeared to influence the work reported in this paper.

## References

[1] Fischbeck K.H. (2016). Spinal and bulbar muscular atrophy overview. J. Mol. Neurosci..

[2] Miura M., Shintaku H., Numasawa Y., Ozaki K., Kanouchi T., Ishikawa K., Yokota T. (2025). An autopsy case of coexisting spinal and bulbar muscular atrophy and multiple system atrophy. Neuropathology.

[3] Oyama G., Hayashi A., Mizuno Y., Hattori N. (2009). Mechanism and treatment of dropped head syndrome associated with parkinsonism. Parkinsonism Relat. Disord..

[4] Hanajima R., Terao Y., Nakatani-Enomoto S., Hamada M., Yugeta A., Matsumoto H., Yamamoto T., Tsuji S., Ugawa Y. (2009). Postural tremor in X-linked spinal and bulbar muscular atrophy. Mov. Disord..

[5] Anagnostou E., Zachou A., Breza M., Kladi A., Karadima G., Koutsis G. (2019). Disentangling balance impairments in spinal and bulbar muscular atrophy. Neurosci. Lett..

